# The long non‐coding RNA HLNC1 potentiates hepatocellular carcinoma progression via interaction with *USP49*

**DOI:** 10.1002/jcla.23462

**Published:** 2020-07-21

**Authors:** Xinye Qian, Shitong Li, Zhoujing Yang, Jun Zhang

**Affiliations:** ^1^ Department of Anesthesiology Huashan Hospital, Fudan University Shanghai China

**Keywords:** hepatocellular carcinoma, HLNC1, HSF1, *USP49*

## Abstract

**Background:**

The hepatocellular carcinoma (HCC) represents a serious malignancy worldwide especially in China. Our transcriptome analysis identifies a novel long non‐coding RNA (lncRNA) termed HLNC1. However, the function of HLNC1 in HCC remains to be determined.

**Methods:**

Novel lncRNAs were screened using lncRNA profiling. Relative expression was quantified by qRT‐PCR. In vitro experiments such as migration and viability assays were performed. In vivo implantation experiments were conducted to investigate tumorigenic functions. RNA‐RNA interaction assay was performed to determine *USP49* as HLNC1 binding partner.

**Results:**

We found that HLNC1 was markedly upregulated in HCC samples and cell lines. HLNC1 could promote viability and migration of HCC cells. Meanwhile, we could also observe an oncogenic effect of HLNC1 in vivo. By RNA‐RNA interaction assay, we unraveled *USP49* transcript as the HLNC1 binding partner. HLNC1‐*USP49* interaction dramatically destabilized *USP49*. Heat‐shock factor 1 (HSF1) was shown to directly induce HLNC1 expression. The therapeutic potential of targeting HLNC1 was investigated using antisense oligonucleotides (ASOs). The ASO construct which significantly depleted HLNC1 expression could strongly attenuate xenograft tumor growth.

**Conclusions:**

Our data suggested that HLNC1 may advance HCC progression and act as a potential target for intervention.

## Graphical abstract

We identified a novel lncRNA termed HLNC1 and HLNC1 was significantly upregulated in HCC samples. The ASO construct which significantly depleted HLNC1 expression could strongly attenuate xenograft tumor growth. HLNC1 may advance HCC progression and act as a potential target for intervention.

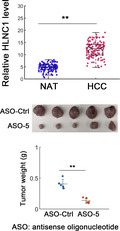

## 1 INTRODUCTION

The hepatocellular carcinoma (HCC) belongs to a common malignancy worldwide especially in China.^1^ Patients with HCC usually suffer from poor prognosis and low 5‐year survival owing to the extreme complexity of clinical situations.^2^ Liver resection and chemotherapy are among the primary strategies during HCC treatments, although the efficiency of such treatments is disappointing.^3^ Another challenge argues that chemotherapeutics sometimes leads to unavoidable cellular survival and possible chemoresistance.^4^ Therefore, identifying the underlying mechanisms of HCC progression and novel therapeutic targets may substantially improve HCC outcomes.

The majority of genome transcribes non‐coding sequences termed non‐coding RNAs. Fraction of these transcripts with >200 nucleotides in length has been defined as long non‐coding RNAs (lncRNAs).^5^ These transcripts are of minimal or no protein‐coding capacities.^6^ To date, numerous lncRNAs have been uncovered and the function of most lncRNAs is still elusive.^7^ A few lncRNAs have been shown to play diverse roles during HCC progression. For example, lncRNA‐ANGPTL1 can interact with integrin α1β1 to suppress downstream JAK/STAT signaling and impairs HCC development.^8^ Additionally, lncRNA HULC may increase the stability of Sirt1 to enhance autophagy and facilitate chemoresistance in HCC cells treated with oxaliplatin, 5‐fluorouracil, or pirarubicin.^9^ A novel lncRNA named uc.134 inhibits LATS1 degradation by suppressing LATS1 ubiquitination to abolish HCC proliferation.^10^ Notably, lncRNA can also sponge miR‐23b‐3p via HOTAIR and advance the epithelial‐mesenchymal transition (EMT) process during HCC progression.^11^ However, the function of a novel lncRNA HLNC1 is still unknown in HCC.

In current work, we identified a novel lncRNA termed hepatocellular carcinoma‐associated lncRNA on chromosome 1 (HLNC1) by lncRNA profiling. We found HLNC1 is significantly upregulated in HCC tissues and human specimens. HLNC1 primarily localizes in the cytoplasm and promotes viability and migration of HCC cells. Meanwhile, HLNC1 also plays an oncogenic role to advance in vivo tumor growth. Mechanistic studies showed that HLNC1 interacts with *USP49* mRNA via its N‐terminal region. HLNC1‐*USP49* interaction dramatically destabilizes *USP49* transcripts. Interestingly, HSF1 is responsible for HLNC1 induction and HLNC1 promoter displays enhanced HSF1 occupancy. The therapeutic effect of HLNC1 is further evaluated using antisense oligonucleotides (ASOs) and ASO‐mediated HLNC1 silence profoundly inhibits HCC growth and metastasis. Collectively, we have shown an oncogenic role for the novel lncRNA HLNC1 in HCC and HLNC1 may serve as a potential therapeutic target for pharmaceutical intervention.

## 2 MATERIALS AND METHODS

### 2.1 Cell lines

293T and HCC cell lines were obtained from Shanghai Institute of Cell Biology. HCC cells were maintained in DMEM with high glucose, 8% FBS, 150 µg/mL penicillin, and 100 U/mL streptomycin. Throughout all the experiments, passage number varied between 3rd and 10th for all cells. For every experiment, it was ensured that cells had been passaged at least thrice after thawing. Cell lines were verified by PCR without contamination by mycoplasma.

### 2.2 Human samples

The HCC samples and paired normal adjacent tissues were collected from the primary sites in patients as previously described from February 2015 to Jun 2018.^12^ Notably, the human specimens were frozen immediately and stored at −80°C refrigerator. Totally, 120 samples were harvested. Experimental procedures related to human samples have been formally approved by Human Research Ethics Committee at Fudan University Shanghai Cancer Center and in accordance with the 1975 Declaration of Helsinki.

### 2.3 Quantitative PCR (qPCR)

Total RNAs were extracted with Trizol reagent, according to the manufacturer's instructions. For lncRNA expression, the first‐strand cDNA was synthesized using Superscript I (Invitrogen) with random primers. qPCR was performed by a miScript SYBR Green PCR Kit (Qiagen). The primers are shown in Table S1.

### 2.4 Short hairpin RNA (shRNA) and overexpression

The lentivirus plasmid for stable RNA interference and the short hairpins targeting HLNC1 (shHLNC1) were designed and purchased from BGI (Beijing). A non‐targeting silencing RNA was used as negative control (shCtrl). Virus packaging was performed in 293T cell after co‐transfection of pLVX lentivirus plasmids using Lipofectamine 2000. To established stable HLNC1 overexpression clones, sub‐confluent cells were transfected with an empty pCMV expression plasmid (control) or pCMV vector containing HLNC1 (HLNC1) using Lipofectamine 2000 (Invitrogen) according to the manufacturer's instructions. After 48‐h transfection, G418 (600 mg/L) was added for selecting stable clones.

### 2.5 Statistical analysis

Statistics were calculated using SPSS (version 16). Data were represented as mean ± SD. Mann‐Whitney test was selected for comparison between two groups and ANOVA was used for detecting significance among more than three groups followed by LSD *post hoc*. At least three replicates were shown. *P* < .05 was statistically significant.

## 3 RESULTS

### 3.1 HLNC1 identification using lncRNA‐seq

**FIGURE 1:**
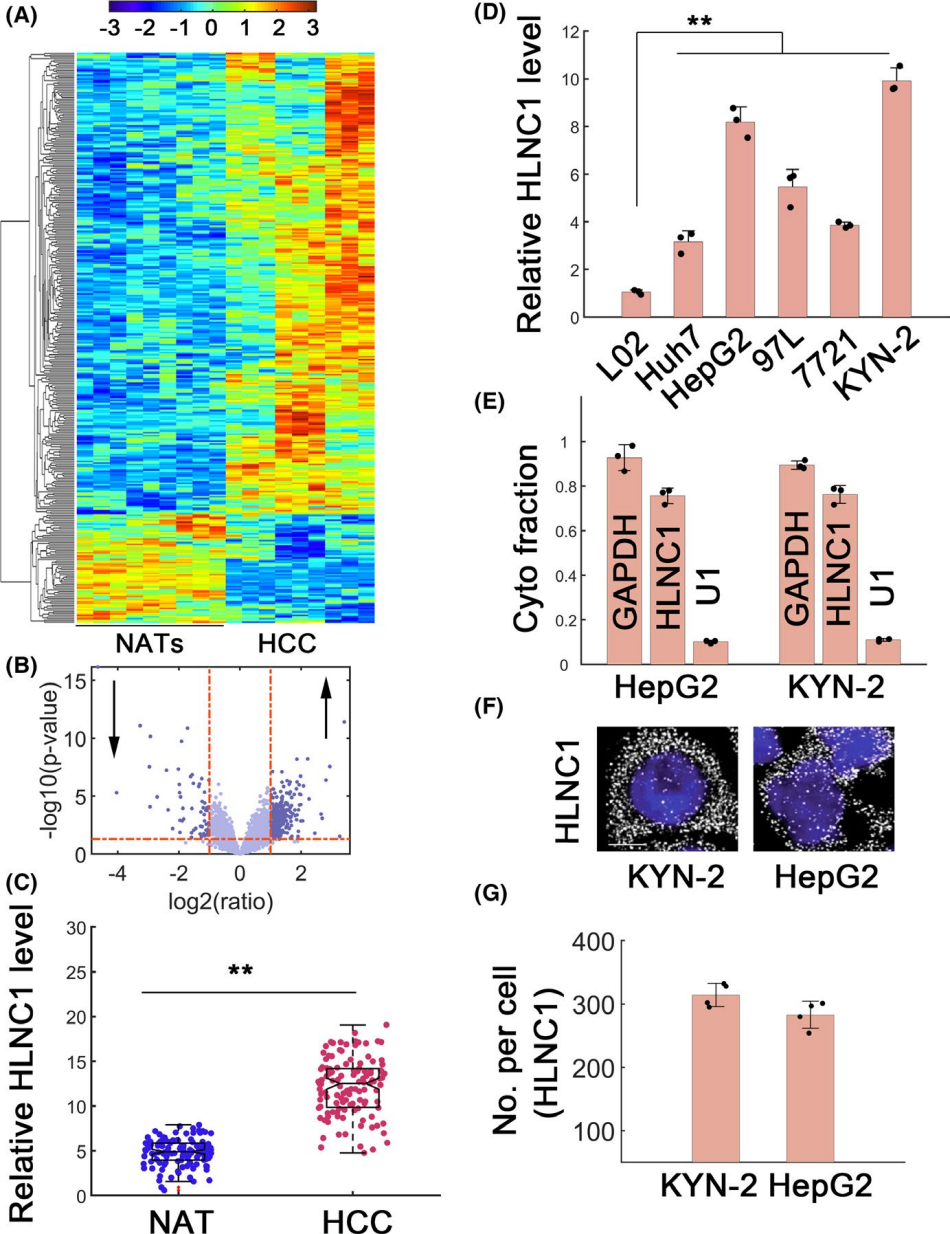
Identifying HLNC1 in HCC. A, RNA‐seq in normal adjacent tissues (NATs) and HCC samples. Nine paired samples were used for sequencing. B, Volcano plot identified significantly altered lncRNAs. The downward and upward arrows indicated significantly downregulated or upregulated lncRNAs, respectively. C, HLNC1 expression in NATs and HCC samples, n = 120. D, HLNC1 abundance in normal L02 cell and five HCC cell lines. E, Subcellular fractionation for HLNC1 in KYN‐2 and HepG2 cells. F, The smFISH to detect HLNC1 molecules in KYN‐2 and HepG2 cells. Scale bar: 5 μmol/L. G, Quantification for (F). **: *P* < .01

To explore HCC‐associated lncRNAs, we performed RNA sequencing (RNA‐seq) in normal and cancerous tissues (Figure 1A,B). Results identified 273 upregulated and 64 downregulated lncRNAs in HCC samples (Figure 1A,B). To investigate lncRNAs associated with cancer progression, we focused on significantly upregulated lncRNAs in HCC samples (Figure 1B, upward arrow). Three novel hits ENSG00000259343 (RP11‐761I4.3), ENSG00000257681 (RP11‐341G23.4), and ENSG00000237950 (HLNC1) were identified (Table S2). Since lncRNA HLNC1 (ENSG00000237950) displayed highest fold upregulation among the three novel ones, we chose it for further analysis (Table S2). The HLNC1 was located on chromosome 1 with 2224 nucleotides (Figure S1A,B). The 2224‐bp transcripts were generated from the reverse strand with two exons (Figure S1B). This transcript showed minimal coding potential as evaluated by CPAT (Figure S1C). HLNC1 was significantly upregulated in HCC samples compared with normal adjacent tissues (Figure 1C, n = 120). Cancerous HCC cell lines also revealed higher HLNC1 expression compared with normal L02 cells (Figure 1D). HLNC1 expression was not markedly correlated with age, gender, cirrhosis status, or capsular formation (Table S3). Instead, HLNC1 is significantly associated with tumor size, metastasis, and TNM stages (Table S3). Subcellular fractionation study demonstrated that HLNC1 was predominantly distributed in cytoplasm (Figure 1E). The single molecule fluorescence in situ hybridization (smFISH) also showed dominant distributions in cytoplasm (Figure 1F). Quantification revealed that there were approximately 300 HLNC1 molecules per cell in KYN‐2 and HepG2 cells (Figure 1G). These data collectively suggested HLNC1 is significantly upregulated in HCC with cytoplasmic localization. Since KYN‐2 and HepG2 cells showed higher HLNC1 abundance, we selected these two cell lines for further assay.

### 3.2 HLNC1 promotes HCC progression in vitro

**FIGURE 2:**
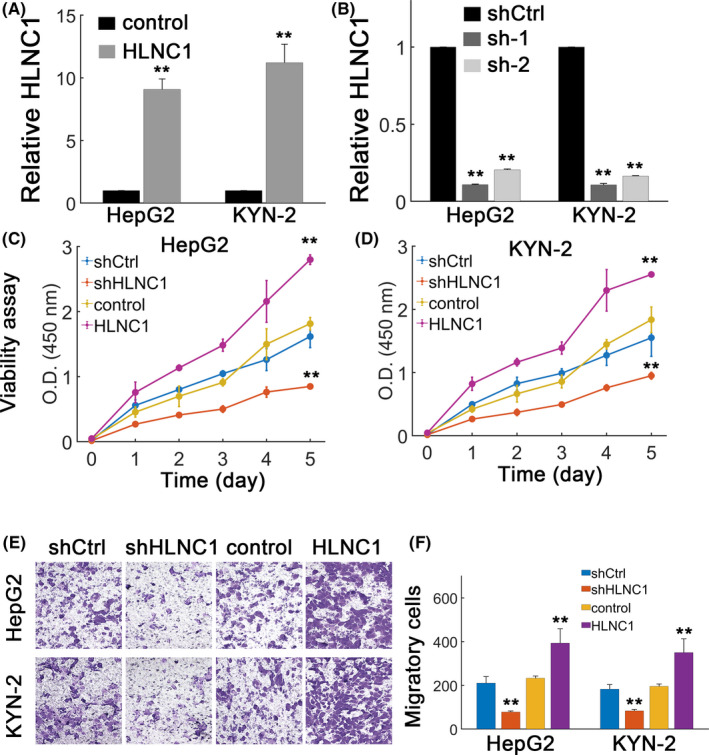
HLNC1 promotes HCC in vitro. A, The HepG2 and KYN‐2 cells transfected with lentiviral control (control) or lentiviral vector carrying HLNC1 (HLNC1). B, HepG2 and KYN‐2 cells were transfected with shCtrl or two shHLNC1 designs (sh‐1 and sh‐2). The sh‐HLNC1‐1 showed higher efficiency and was selected thereafter. C‐D, Viability assay for HepG2 (C) and KYN‐2 (D) cells transfected with shCtrl, shHLNC1, lentiviral control (control), or lentiviral vector carrying HLNC1 (HLNC1). E, Cellular migration assay for HepG2 and KYN‐2 cells transfected with shCtrl, shHLNC1, lentiviral control (control), or lentiviral vector containing HLNC1 (HLNC1). F, Quantification results for (E). **: *P* < .01

Since HLNC1 was significantly upregulated in HCC cell lines and tissues, we then explored the effect of HLNC1 in vitro. Stably transfected HepG2 and KYN‐2 cell lines were generated to significantly overexpress HLNC1 (Figure 2A) or knock down HLNC1 levels (Figure 2B). HLNC1 overexpression markedly increased HepG2 viability whereas HLNC1 silence dramatically decreased the viability of HepG2 cells (Figure 2C). Qualitatively similar results could be obtained in KYN‐2 cells (Figure 2D). Meanwhile, HLNC1 overexpression potentiated cellular migration in HepG2 and KYN‐2 cells (Figure 2E,F). HLNC1 depletion instead strongly inhibited migratory capacity (Figure 2E,F). These results suggested that HLNC1 promoted HCC malignancy in vitro.

### 3.3 The in vivo tumor growth is promoted by HLNC1

**FIGURE 3:**
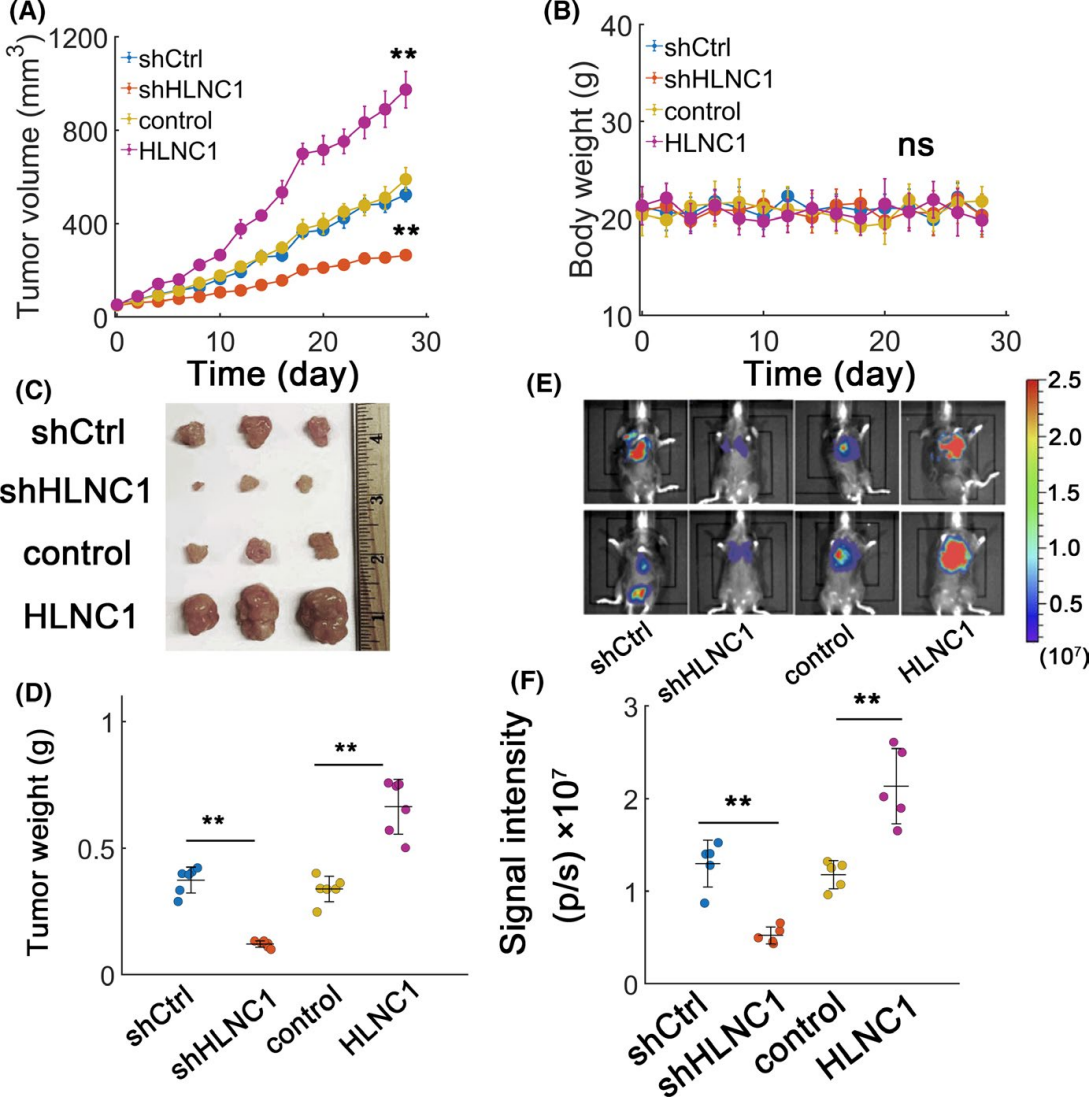
HLNC1 potentiates tumor growth in vivo. A, Tumor volume changes for 4 wk in HepG2 xenografts transfected with shCtrl, shHLNC1, lentiviral control (control), or lentiviral vector carrying HLNC1 (HLNC1). B, Body weights alterations for mice as in (A). C, Xenograft tumor images in HepG2 xenografts with either HLNC1 silence or overexpression. D, Quantification for (C). E, Bioluminescence imaging (BLI) for a metastatic model. F, The fluorescence signal intensities for (E) with either HLNC1 depletion or overexpression in HepG2 cells. **: *P* < .01

Since HLNC1 has an oncogenic role in vitro, we anticipated that HLNC1 might also exhibit an oncogenic effect in vivo. We found that HLNC1 overexpression indeed resulted in an expansion of tumor volumes whereas decreasing HLNC1 played an inhibitory effect (Figure 3A). Meanwhile, we noted that HLNC1 did not change the mice body weights significantly (Figure 3B). The xenograft growth was potentiated with HLNC1 overexpression and consistently suppressed when HLNC1 was depleted (Figure 3C,D). Meanwhile, HLNC1 overexpression also promoted metastasis as observed in BLIs (Figure 3E,F). HLNC1 silence attenuated the metastatic effect (Figure 3E,F). These results collectively indicated that HLNC1 promoted in vivo tumor growth.

### 3.4 HLNC1 interacts with and stabilizes *USP49* transcript

**FIGURE 4:**
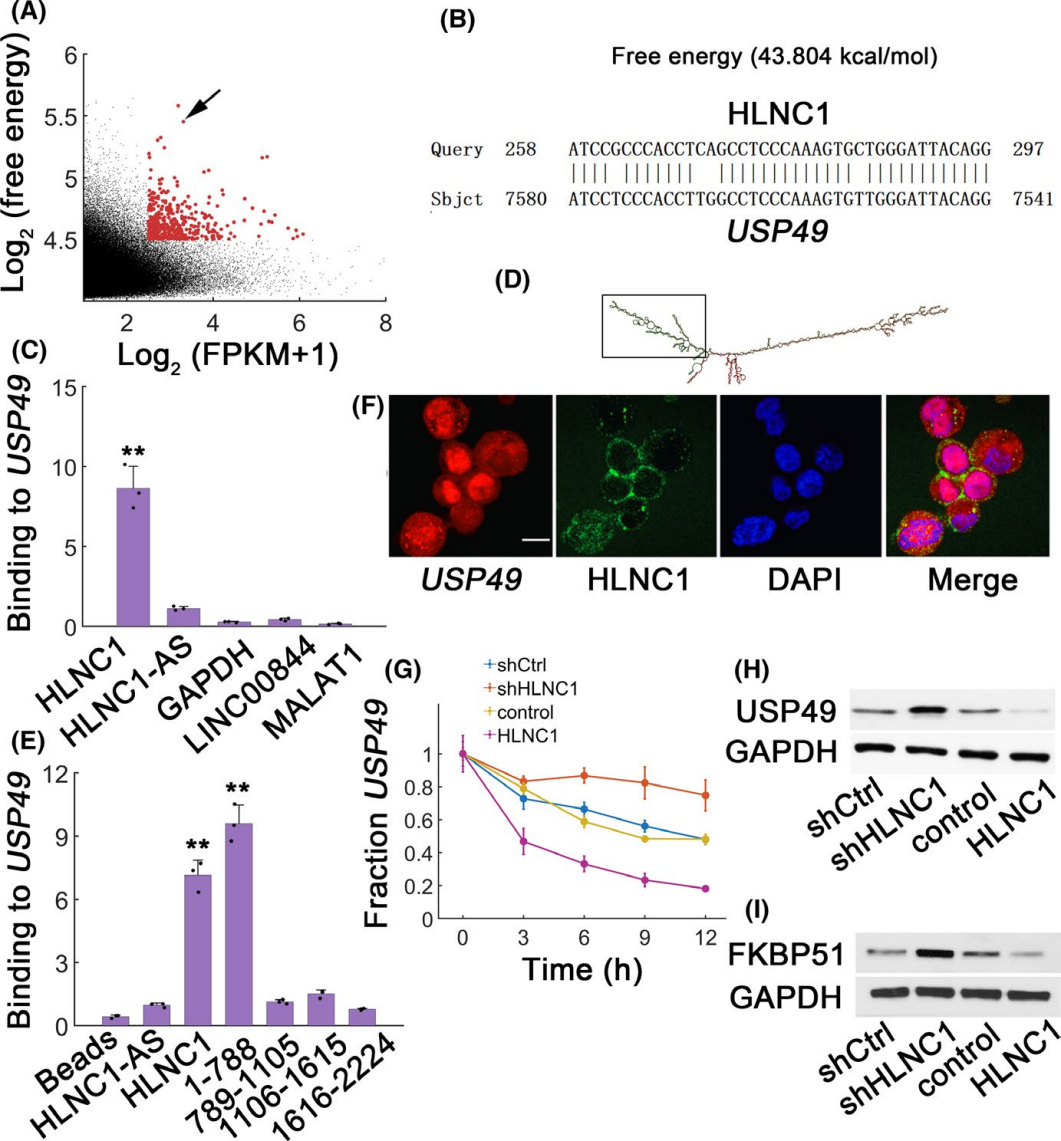
Co‐localization between HLNC1 and *USP49* transcript. A, *P*rediction of HLNC1‐binding partners. y: log_2_‐absolute RNA binding energy between HLNC1 and putative targets; x: log_2_‐average expression of targets. B, Predicted RNA‐RNA interaction region between HLNC1 and *USP49*. C, In vitro RNA‐RNA binding assay for HLNC1 and *USP49* (antisense HLNC1, HLNC1‐AS, *GAPDH,* and non‐target lncRNA MALAT1 and LINC00844). D, Predicted HLNC1 secondary structure via the online tool RNAfold (http://rna.tbi.univie.ac.at//cgi‐bin/RNAWebSuite/RNAfold.cgi). The structure in the box indicated the interaction domain with *USP49*. E, In vitro RNA‐RNA binding assay identified HLNC1 binding domain. Normalization was to HLNC1‐AS control. F, FISH for *USP49* transcript (red), HLNC1 (green), and DAPI (blue). Scale bar: 50 μmol/L. G, The qPCR for *USP49* in HepG2 cells transfected with shCtrl, shHLNC1, control, or HLNC1 lentiviral vector. (H‐I) USP49 (H) and (I) FKBP51 protein expression in HepG2 cells transfected with shCtrl, shHLNC1, control, or lentiviral vector carrying HLNC1 48 h after transfection. **: *P* < .01. The black dots showed measurements

Recent work has demonstrated that RNAs can interact with target mRNAs via RNA‐RNA association.^13^ To determine putative HLNC1 targets, ntraRNA was utilized to implement an unbiased prediction.^14^ Results showed that *ubiquitin carboxyl‐terminal hydrolase 49* (*USP49*) mRNA was a putative target for HLNC1 binding (Figure 4A). In vitro RNA‐RNA assay confirmed HLNC1‐*USP49* interaction (Figure 4B,C, *GAPDH*, lncRNA MALAT1, and LINC00844 were all used as negative controls). The secondary structure was predicted via online tool RNAfold (Figure 4D). Then, various HLNC1 truncates were generated to locate the interaction domains by in vitro RNA‐RNA assays (Figure 4E). We found that the N‐terminal region of HLNC1 was responsible for *USP49* binding (Figure 4E). FISH data identified significant co‐localization of HLNC1 and *USP49* mRNA (Figure 4F). Notably, HLNC1 significantly destabilized *USP49* transcripts (Figure 4G). As a result, the USP49 protein levels were consistently downregulated with HLNC1 overexpression (Figure 4H). Previous work showed that USP49 can deubiquitinate and stabilize FKBP51.^15^ As expected, FKBP51 levels were also reduced by HLNC1 overexpression (Figure 4I). These results suggested that HLNC1 interacted with and destabilized *USP49* mRNA.

### 3.5 HSF1 regulates HLNC1 expression

**FIGURE 5:**
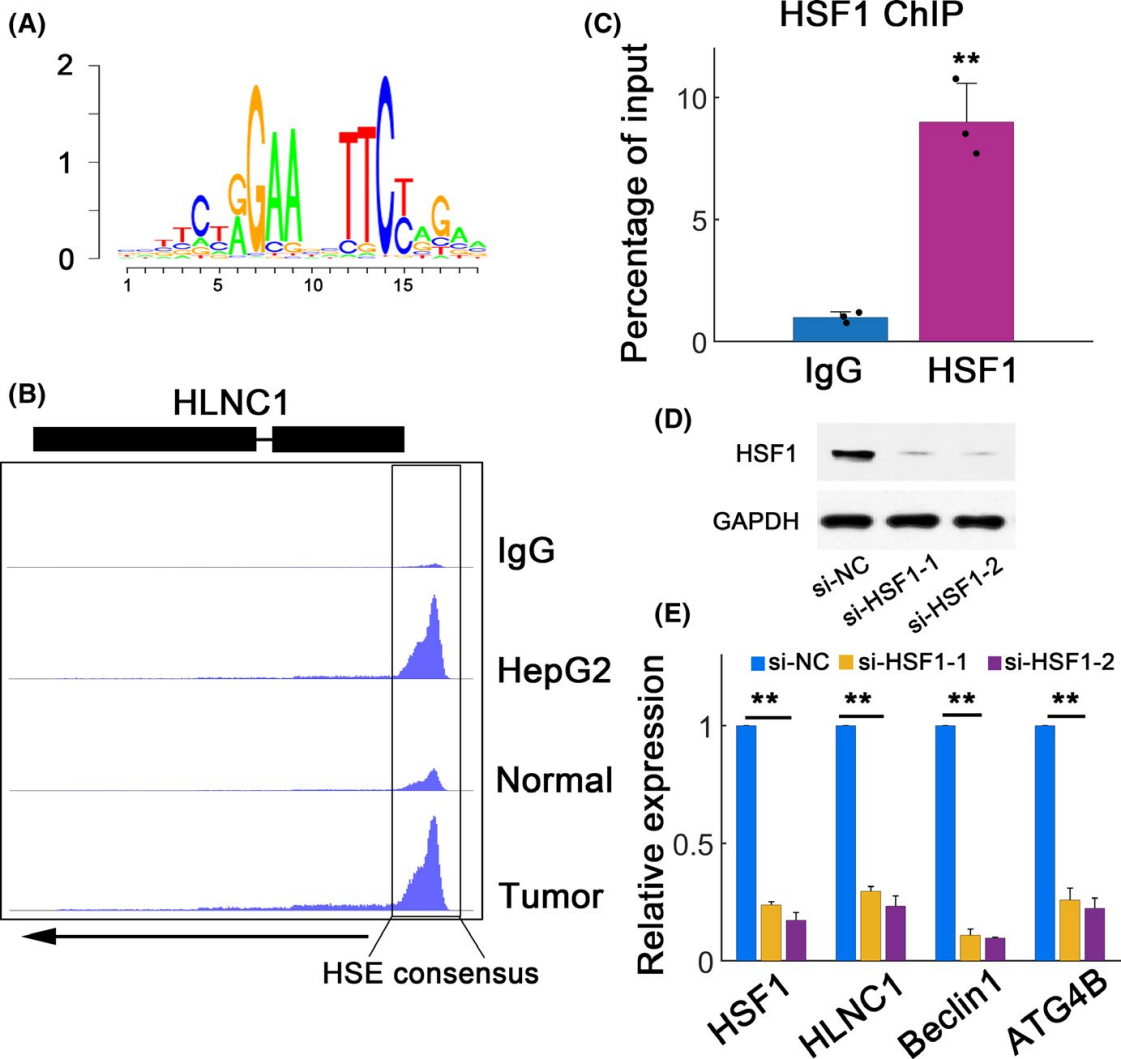
HLNC1 is directly induced by HSF1. A, A canonical HSE motif. HSE: heat‐shock element. B, HSF1 ChIP‐seq data in HepG2 cells, normal adjacent tissues, and HCC tissues. Enrichment was normalized. Control (IgG) or HSF1 ChIP‐seq data around HLNC1 locus. C, ChIP‐qPCR in HepG2 cells showing HSF1 or IgG enrichment scores around HLNC1 promoter sequences. D, Two RNA silencing designs for *HSF1* with siRNA negative control (si‐NC) and two specific siRNAs targeting *HSF1* (si‐*HSF1‐1* and si‐*HSF1‐2*). E, Expression of HSF1 and its target genes (*Beclin1* and *ATG4B*) transfected with si‐NC or two *HSF1*‐specific siRNAs. n = 3 independent experiments. **: *P* < .01

To identify the potential transcription factor responsible for HLNC1 induction, motif research was conducted.^16^ The results showed canonical heat‐shock element (HSE) consensus around the promoter regions of HLNC1 (Figure 5A). Since heat‐shock factor 1 (HSF1) positively contributes to HCC progression,^17^ we further explored the HSF1 occupancy at HLNC1 locus. The (chromatin immunoprecipitation sequencing) ChIP‐seq data further demonstrated significantly enriched peaks in HepG2 and HCC samples around the HLNC1 promoters (Figure 5B). ChIP‐qPCR was performed in HepG2 cells and showed significantly high HLNC1 abundance (Figure 5C). We then investigated how HSF1 silence affected the expression of HLNC1 using two si‐*HSF1* siRNAs (si‐*HSF1‐*1 and si‐*HSF1*‐2, Figure 5D). Both siRNAs substantially decreased HSF1 expression (Figure 5D). *HSF1* depletion therefore led to dramatic decrease in *HSF1*, HLNC1, as well as two HSF1 downstream target genes^18^ (Figure 5E). These observations suggested that HSF1 directly regulates HLNC1 expression.

### 3.6 HLNC1 may serve as a putative therapeutic target

**FIGURE 6:**
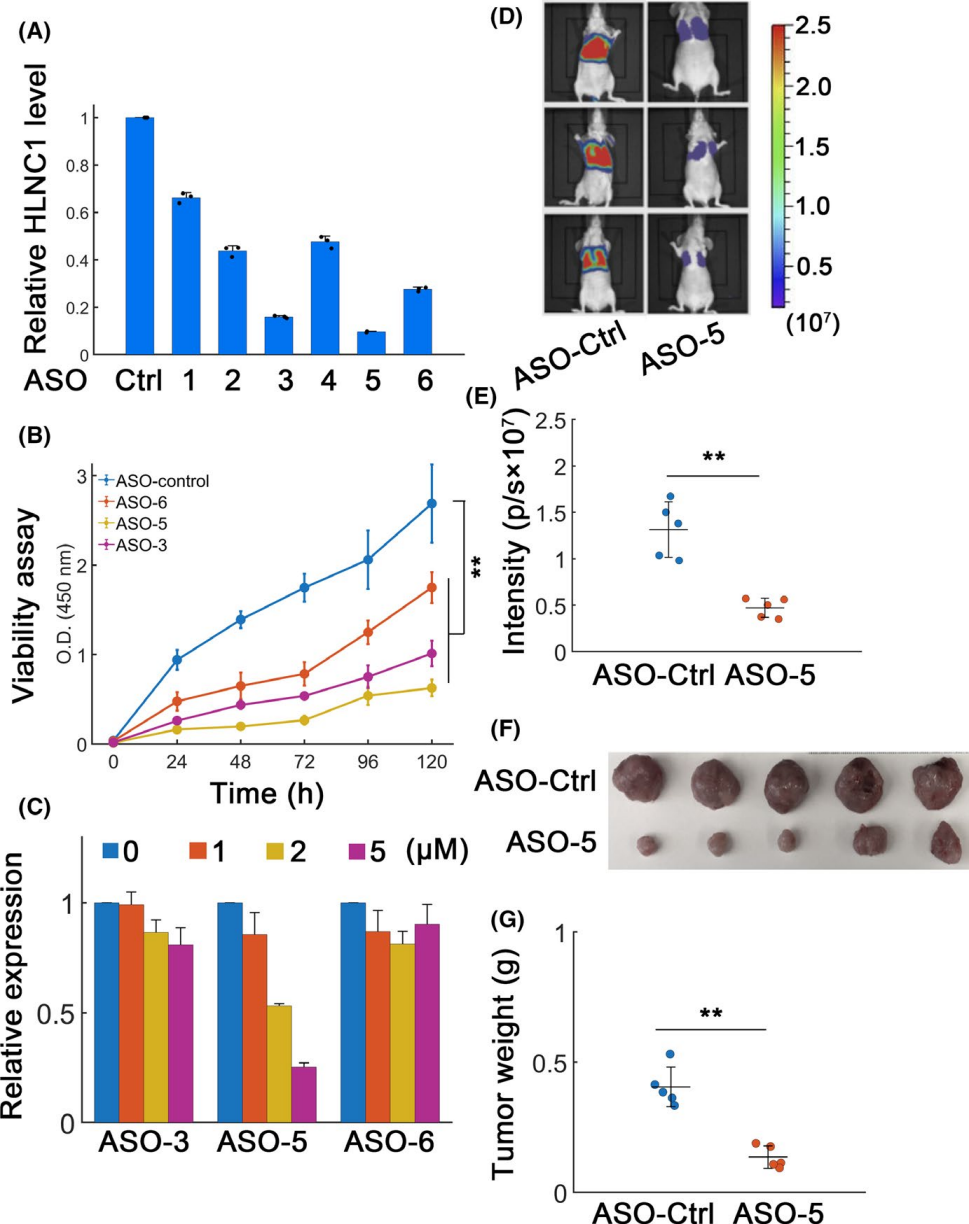
Therapeutic potential for ASO targeting HLNC1. A, The qRT‐PCR results for relative HLNC1 expression in HepG2 cells treated with the control ASO (ASO‐control) or distinct ASO constructs. B, ASO‐control or different ASOs targeting HLNC1 on HepG2 cell viability. C, Free uptake assays in HepG2 cells using different concentrations of ASOs (0, 1, 2, and 5 μmol/L). The relative HLNC1 abundance was measured. D, Metastasis model. 20 d after implantation, mice were randomly assigned into two groups (ASO‐Ctrl or ASO‐5). The ASO‐Ctrl or ASO‐5 treatment was conducted every 2 d immediately after the group assignment. E, Quantification of fluorescence signals for (D). F, Xenograft tumor images for HepG2 cells treated with ASO‐Ctrl or ASO‐5. After 20 d of HepG2 implantation, all mice were randomly divided into two groups with similar starting tumor burdens (ASO‐Ctrl and ASO‐5). Then, ASO‐Ctrl and ASO‐5 treatments were immediately applied every 2 d. G, Statistics for (F). **: *P* < .01

We next explored whether HLNC1 may behave as a potential target using antisense oligonucleotides (ASOs). Six ASOs were designed and qRT‐PCR data showed that all ASOs significantly decreased HLNC1 expression in HepG2 cells (Figure 6A). Since ASO‐3, ASO‐5, and ASO‐6 displayed relatively higher efficiency, they were further evaluated on HepG2 viability. Results demonstrated that ASO‐3, ASO‐5, and ASO‐6 dramatically decrease HepG2 cell viability (Figure 6B). Then, HepG2 cells were treated with ASOs without transfection agents to mimic the in vivo conditions. Results suggested that ASO‐5 was more effective in free uptake assays (Figure 6C) and was chosen for further assay. An in vivo metastasis model was performed. We found that ASO‐5 significantly reduced metastasis compared with ASO‐Ctrl‐ group (Figure 6D,E). ASO‐Ctrl or ASO‐5 treatment did not significantly affect mice body weight (Figure S2A). The biochemical parameters such as aspartate aminotransferase (AST) and alanine aminotransferase (ALT) levels were similar in ASO‐Ctrl‐ or ASO‐5‐treated groups (Figure S2B). Meanwhile, xenograft models were carried out and results suggested that ASO‐5 could dramatically inhibit tumor growth (Figure 6F,G). Overall, these results implied that HLNC1 might be used as a potential target for therapeutics in HCC.

## 4 DISCUSSION

In this work, we identified a novel lncRNA named HLNC1 and evaluated its role in hepatocellular carcinoma. HLNC1 transcription was dramatically elevated in hepatocellular carcinoma tissues and cell lines. HLNC1 displayed significantly oncogenic effects in vitro and in vivo. Mechanistically, HLNC1 interacted with *USP49* mRNA to increase *USP49* turnover. HSF1 was shown to directly activate HLNC1 transcription. Therefore, we identified a novel HSF1‐HLNC1‐*USP49* axis during hepatocellular carcinoma progression. Targeting the HSF1‐HLNC1‐*USP49* axis by specific antisense oligonucleotides for HLNC1 has presented a promising effect to eradicate HCC cells.

HSF1 is a conserved helix‐turn‐helix transcription factor which is activated when cells encounter heat‐shock, chemical agents, or other forms of environmental stresses.^17^, ^19^ HSF1 is the most characterized isoforms among the six HSF gene products.^20^ The trimerized HSF1 may translocate to the nucleus and then binds the HSE of many HSPs (heat‐shock proteins).^21^ The enhanced expression of specific HSPs has been evident in many cancers, and therefore, HSPs may play oncogenic roles during cancer progression.^22^ Cancer‐associated fibroblasts (CAFs) show hyperactivated HSF1, which reprograms tumor stroma and promotes tumor malignancy.^23^ HSF1 can promote metastasis in HCC and potentially serve as a therapeutic target.^17^ The oncogenic function of HSF1 is also observed in osteosarcoma, breast cancer, colon, melanoma, and pancreatic cancer.^20^, ^24^ As a result, the role of HSF1 in oncogenesis is emerging. The lncRNAs such as NEAT1, lincRNA‐ROR, EFNA3, and GAS5 are HSF1 targets, which are all involved in cancer.^25^, ^26^ In current work, we have extended the wiring diagrams in HSF1 network by identification of HLNC1 as a novel HSF1 direct target. We found that HSF1 could bind to the promoter region of HLNC1 and HSF1 silence decreased HLNC1 abundance. ChIP‐qPCR also verified HSF1 binding to HLNC1 promoter. Further studies are required to enrich our understanding about the role of lncRNA in heat‐shock responses and cancer progression.

The USP49 has been identified as a tumor suppressor protein which participates in DNA damage response via p53‐positive feedback loop.^27^ USP49 can enhance the transcriptional activity and decrease the protein turnover for p53 protein.^27^ Furthermore, USP49 can also improve chemoresistance and inhibit tumor progression by mediating FKBP51‐AKT signaling.^15^ The FKBP51 can be stabilized by USP49 via increased deubiquitylation.^15^ As a result, maintaining the level of USP49 plays a critical role in tumor suppression. In current work, we have found that a novel lncRNA termed HLNC1 could regulate *USP49* mRNA stability. The direct USP49 and HLNC1 binding strongly raised the degrading rate of *USP49* transcripts whereas silencing HLNC1 had an opposite effect. The upregulation of HLNC1 in HCC through HSF1 may partially enhance cancer progression via decreasing the level of *USP49* mRNA and USP49 proteins were consistently downregulated. Therefore, the novel HSF1‐HLNC1‐*USP49* axis may add a novel layer of complexity in HCC.

Given the oncogenic role of HLNC1, the therapeutic effect of HLNC1 targeting is potentially beneficial for HCC inhibition. Recently, ASOs have been demonstrated to be an effective strategy to target RNA in vivo.^28^ In addition, modifying ASOs with phosphorothioate has been reported to enhance the efficiency to eradicate prostate cancer cells.^29^ Meanwhile recently, an ASO targeting IONIS‐APO(a)_Rx_ has already been shown to alter apolipoprotein expression to treat calcific aortic valve stenosis (CAVS) in a clinical trial.^30^ Interestingly, several ASOs were designed in current work which significantly attenuate HLNC1 expression (especially the ASO‐3 and ASO‐5 constructs). Furthermore, these ASOs can diminish in vivo xenograft tumor growth without significant side effects. Further improvement on ASO uptake efficiency without toxicity should be resolved in future.

In conclusion, we have identified a novel lncRNA HLNC1 with potential oncogenic function. HSF1 contributes to HLNC1 induction which then destabilizes *USP49* transcripts leading to HCC progression. The novel HSF1‐HLNC1‐*USP49* axis may serve as a target for pharmacological intervention to eradicate HCC cells.

## Supporting information

Supplementary Material

## Data Availability

The data that support the findings of this study are available from the corresponding author upon reasonable request.
