# Supplementary material for: The long non‐coding RNA HLNC1 potentiates hepatocellular carcinoma progression via interaction with USP49
Source: J Clin Lab Anal. 2020 Jul 21;34(11):e23462. doi: 10.1002/jcla.23462 (PMC7676201; doi:10.1002/jcla.23462)
Supplement: Supplementary file 1 — Supplementary Material [file JCLA-34-e23462-s001.docx]

**SUPPLEMENTARY DATA**

**SUPPLEMENTAL METHODS**

**RNA sequencing (RNA-seq)**

Total RNA was prepared from tumor tissues excised using the Trizol RNA-extraction protocol with subsequent purification of RNA using RNeasy kit (Qiagen). Total RNA was assessed for quality using an Agilent Tapestation. RNA libraries were generated from 5 μg of RNA using Illumina TruSeq Stranded mRNA Sample Prep Kit following the manufacturer’s instructions. RNA libraries were multiplexed and sequenced with 50 base pair (bp) single end reads (SR50) on an Illumina HiSeq3000 system.

**ChIP-seq**

The ChIP-seq was conducted using external datasets (GSE54503, GSE60984 and GSE31477). The genomic reads were first aligned to GRCh38 genome reference using STAR protocols. Unpaired and primary alignments were excluded. The enrichment plot was obtained from BigWig by smoothing splines [1]. The baseline signals were obtained from a window with longest distance from transcription start site (TSS).

**Single molecule fluorescence *in situ* hybridization (smFISH)**

The smFISH was conducted as previously described [2]. The probes for HLNC1 and *GAPDH* were designed by and purchased from Qiagen. HepG2 cells were seeded in 12-well plates and incubated for 24 h. After reseeding into 6-well plates, HepG2 cells were fixed in formaldehyde for smFISH (18 h).

**RNA-RNA *in vitro* interaction**

20 μL Protein A/G Magnetic Beads (Sigma) were washed twice using RIP buffer (Millipore) and then incubated with BrU antibody for 1 h at room temperature. After conjugation, beads were further washed twice with RIP wash buffer and then resuspended in buffer containing 30 mM EDTA (Millipore) and RNase Inhibitor (Millipore). 20 pmol of BrU-labeled RNAs were incubated with beads in buffer for 2 h. During incubation, 3 pmol *USP49* RNA fragments were appended into individual tubes and incubation was performed overnight at 4℃. Beads were digested with proteinase K buffer to recover RNAs with RIP Wash Buffer, 1% SDS (Millipore) and 1.2 μg/μl proteinase K (Millipore) at 50°C for 20 min. RNAs were extracted using miRNeasy kit (Qiagen), followed by reverse transcription with Superscript III (Invitrogen). The amount of recovered *USP49* fragments were evaluated by qPCR. Normalization was done by *ΔC_t_* method. ASOs disrupting the interaction between *USP49* and HLNC1 were designed and obtained from IDT.

**Fluorescence *in situ* hybridization (FISH)**

Fluorescence conjugated probes for HLNC1 and *USP49* were designed by and obtained from BGI (Beijing). The non-denaturing conditions were applied to treat samples. HepG2 cells were fixed in 4% formaldehyde and permeabilized with 0.5% Triton X-100 for 10 min, washed with PBS for triplicates and twice in 2×SSC buffer. Then, the DIG-conjugated probes were used for hybridization. Samples were counterstained with DAPI and visualized by a confocal microscopy.

**Subcellular fractionation**

Cytoplasmic and Nuclear RNA Purification Kit (Norgen) was used according to the manufacturer’s guidelines.

***In vivo* tumorigenesis**

2×10^6^ HepG2 cells were resuspended in 150 μl PBS and Matrigel solutions. HepG2 cells were subcutaneously injected into mice (female NOD/SCID mice, 4~5 weeks old). The volumes were evaluated by the formula *V*=*R*×(*r*/2)^2^ where *R* is the longest diameter and *r* is the smallest diameter. For the metastatic model, 4~5-week-old female mice were injected with stably transfected 5×10^6^ HepG2 cells at the lateral tail vein (100 μl). Mice were housed at 20℃ at equal light/dark cycle with free access to water and food. After euthanization for mice, solid tumors were resected and weighed. The bioluminescence imaging (BLI) was shown and quantified using Lumazone imaging system (MAG BioSystems). The experimental protocols were approved by the Institutional Animal Care and Use Committee in Fudan University Shanghai Cancer Center.

**Western blot**

Cells were lysed in RIPA lysis buffer (V900854, Sigma) with protease inhibitor cocktail (Sigma). Protein concentrations were determined by DC protein assay (BioRad) and lysates were boiled. The protein extracts were subject to SDS-PAGE separation with polyvinylidene difluoride membrane (Qiagen). The membranes were blocked in blocking buffer (4% milk in 0.3% TBST) and incubated overnight in a refrigerator with specific primary antibodies. After being washed by TBST for three times, membranes were coated with HRP-conjugated secondary antibody.

**Migration assay**

Serum-free 2×10^5^ cells were loaded in the top chamber of 12-well plate (BD Bioscience) for Transwell migration assays. The medium with 7% serum was added at bottom chamber. After incubation for 12 h, cells migrating into the opposite chambers were fixed with 4% paraformaldehyde (Sigma) and stained with 0.4% crystal violet (Sigma).

**Viability assay**

The Cell Counting Kit-8 (CCK-8, Dojindo, Japan) was used following the manufacturer’s instructions. Briefly, HepG2 and KYN-2 cells were first resuspended and then loaded into twelve-well plates (5×10^5^ cells/well) for 5 days. 30 μl CCK-8 solutions were appended into the cultures. Optical density (450 nm) was quantified using a Spectramax M5 microplate monitor (Molecular Devices).

**Antisense oligonucleotides (ASOs)**

Totally, six ASOs were designed by and purchased from GeneChem (Beijing). An ASO with non-targeting effect was used as an ASO-control. To deliver ASO *in vitro*, 2×10^5^ HepG2 cells were loaded into six-well plates followed by transfection with 1.8 μg ASOs mixed with 6 μl Lipofectamine 2000 and the RNAs were harvested 24 hrs later. For *in vivo* free uptake, 2×10^5^ HepG2 cells were loaded into six-well plates and then transfected with ASO-control or six ASO constructs (ASO-1 to ASO-6) with a gradually increasing concentration (0, 1, 2 and 5 μM) without Lipofectamine 2000. ASO delivery was conducted by intraperitoneal injection every two days at 35 mg/kg *in vivo*.

**Chromatin immunoprecipitation**

2×10^6^ cells were treated with 2% formaldehyde to generate 100 to 500 bp fragments in lysis buffer. Then, beads were washed three times with the lysis/wash buffer followed by being washed with tris-EDTA buffer once. The immunoprecipitants were then eluted and cross-linked for 6 h. The immunoprecipitants were treated with RNase A and proteinase G, then the DNA fragments were subject to chloroform and isoamyl alcohol followed by qRT-PCR.

**Table S1.** Primers and antibodies

| **Name** | **Sequences** |
| --- | --- |
| HLNC1-F | ACCCACATTGTCCACATACCCTTAC |
| HLNC1-R | TCAAACTATACAAATAAAAAAAATG |
| Antisense-F | CCGGCACTGTTTCTGGCATA |
| Antisense-R | GCATTTTACATAATACACGAAAGCA |
| GAPDH-F | CAGTCAGTCACGCGAGGAT |
| GAPDH-R | GTGACGCGGAGAGACAGT |
| U1-F | CAAACTAACAACTCAAGGATCTCCAC |
| U1-R | ACC AAAGTCAAATCTTCCCGTA |
| USP49-F | ACCTCAGGTCAACAACTCGTTTGTC |
| USP49-R | TTATTGCTCCTGGATGCAAGCA |
| HLNC1 1-788-F  -R | TTTTAGAGGGCGGACTGTGT  AAGATGGAGCAGTGAACAGCA |
| 789-1105-F | AATACGACTCACTATAGGGAGAGAAG |
| -R | CACTATAGGGAGAGGGGCAA |
| 1106-1615-F | TAATACGACTCACTATAGGGAG |
| -R | AGTTTCACTGTGTTAGCCAGGAT |
| 1616-2224-F | CAATTATACATAGGAAAACAACG |
| -R | GAGAGAGAATGTGGAAAGCAGC |
| ATG4B-F | GGATCCATGTCGCTTTCTAACAAGCT |
| ATG4B-R | GGAATTCTCAATGAAAGCGGAGGT |
| HSF-1-F | AAATGGATGCGACTTCCGC |
| HSF-1-R | CGTTTGGCTTCAATGTTACC |
| MALAT1-F | GGAGCTAGGAACAGCGAAGCACTA |
| MALAT1-R | TAAGTCTGTACCGCAAAAGAGCTAGGAGC |
| LINC00844-F | TGCAGGGCCCTTACTGCATGACCT |
| LINC00844-R | ATGTCTTCGTGAGCTGGCAGACG |
| Beclin1-F | GGATTCCCTTAGCTTGCCATGCG |
| Beclin1-R | AAATGAAGCTCAGAATATGAAT |
| shHLNC1 #1 | ATAGGTACCGAGCTCTACGGCGTCTTACGATAACGCTGGCCTCGT |
| shHLNC1 #2 | CTTACTTAGATCCTGTGGTCAGTAGCAGAGTGCAGATCAAGTACGG |
| shUSP49 #1 | TGAAGCTTATGTATGCCTAAATTGCCCTTCGCTTTCGAGT |
| shUSP49 #2 | AATGCCAGTAATTCTTAGTATCTGCAGCCTCTTCATTCTAATTCT |
| **ASO No.** | **Sequences** |
| ASO-1 | mUmAmGmVmGATGCAGTGACTmGmTmGmGmG |
| ASO-2 | mUmGmAmUmGGATGAATCAmAmCmUmCmA |
| ASO-3 | mUmGmAmAmGGGCGTATATmGmAmUmGmU |
| ASO-4 | mAmCmUmUmGTTCACATTTAmGmUmAmCmC |
| ASO-5 | mGmUmAmCmATAGGCGTACAmUmGmAmUmU |
| ASO-6 | mGmAmGmUmCACTAGATTCGmUmGmGmCmU |
| ASO-Control | mUmCmUmUmCCGACTAGGTTmCmCmAmUmC |
| **Antibodies or reagents (Catalog NO.)** | **Sources** |
| USP49 (HPA030255) | Sigma |
| GAPDH (G8795) | Sigma |
| HRP secondary antibody  (SAB5300168) | Sigma |
| DAPI (#D9542) | Sigma |
| Biotin RNA Labeling Mix (#11685597910) | Roche |
| HSF1 (SAB2107859) | Sigma |
| hematoxylin (H9627) | Sigma |
| eosin (E4009) | Sigma |
| formaldehyde (47675) | Sigma |
| FKBP51(14155-1-AP) | Proteintech |

| ID | LncRNA names | Log_2_FC (Signal intensity) | *p* value |
| --- | --- | --- | --- |
| ENSG00000237950 | *HLNC1* | 4.7912 | 0.0001 |
| ENSG00000259343 | *RP11-761I4.3* | 3.1796 | 0.0029 |
| ENSG00000257681 | *RP11-341G23.4* | 2.8761 | 0.0043 |

**Table S2:** Significantly upregulated novel lncRNAs

**Table S3:** Correlation between clinicopathological features and HLNC1 levels.

|  |  | HLNC1 expression | |
| --- | --- | --- | --- |
| Features | No. | Low/High | *P* value |
| **Age** |  |  |  |
| < 60 | 62 | 28/34 | 0.181 |
| ≥ 60 | 58 | 32/26 |  |
| **Tumor grade** |  |  |  |
| Well | 35 | 25/10 | <0.001 |
| moderate | 48 | 25/23 |  |
| Poor | 37 | 10/27 |  |
| **TNM stage** |  |  |  |
| I/II | 65 | 43/22 | <0.001 |
| III/IV | 55 | 17/38 |  |
| **Cirrhosis** |  |  |  |
| Present | 53 | 26/27 | 0.500 |
| Absent | 67 | 34/33 |  |
| **Tumor size** |  |  |  |
| < 4 cm | 53 | 35/18 | 0.002 |
| ≥ 4 cm | 67 | 25/42 |  |
| **Metastasis** |  |  |  |
| Absent | 58 | 39/19 | <0.001 |
| Present | 62 | 21/41 |  |
| **Capsular formation** |  |  |  |
| Absent | 82 | 44/38 | 0.163 |
| Present | 38 | 16/22 |  |

TNM: tumor (T), the extent of spread to the lymph nodes (N), and the presence of metastasis (M) (* P < 0.05, ** *P* <0.01). The median value was used as the cut-off.


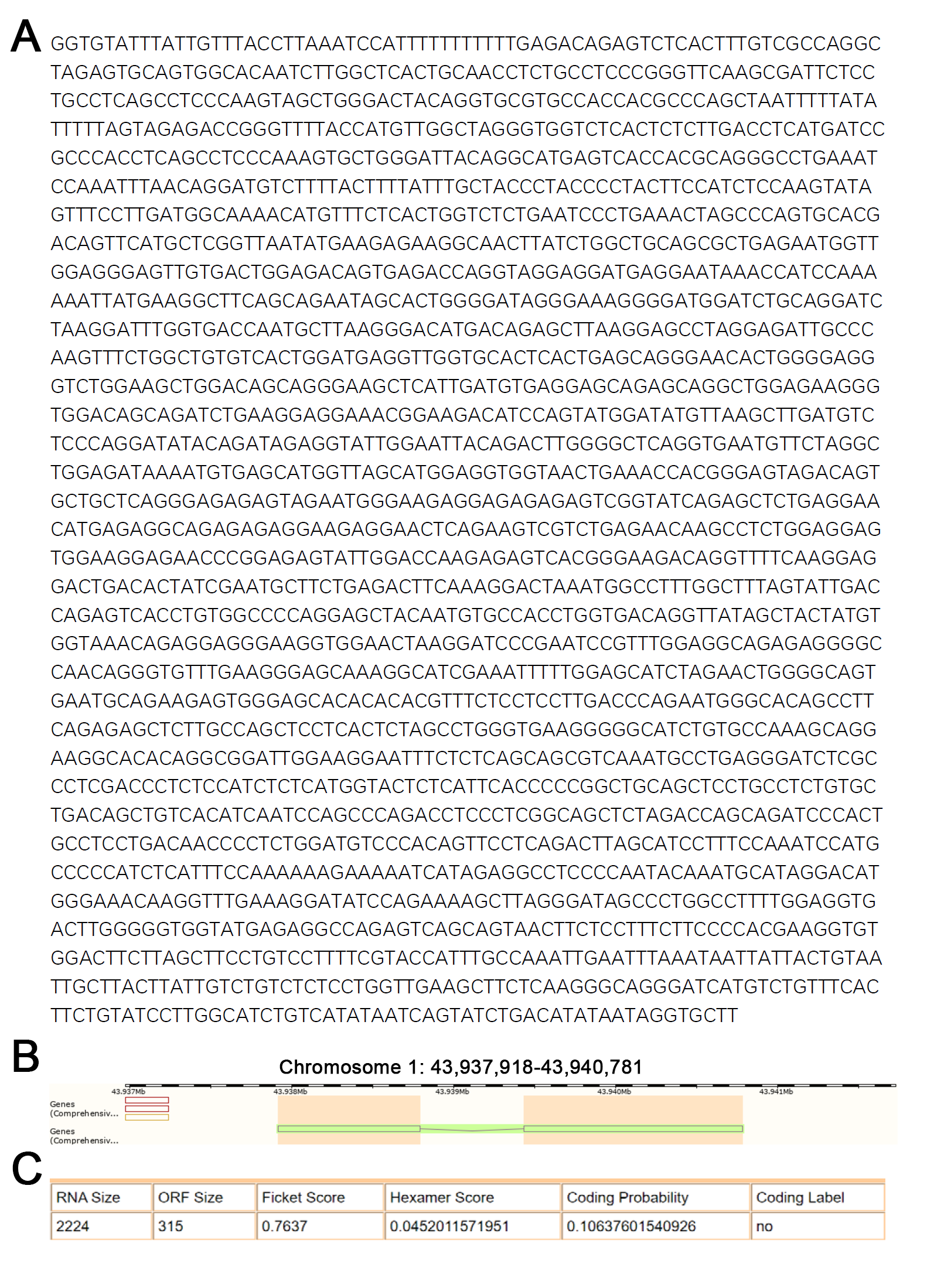


Figure S1. Characteristics of HLNC1. (A) The sequences of full-length HLNC1 (2224 nt). (B) The chromosome location of HLNC1 with two exons. (C) Coding potential for HLNC1 using the online Coding Potential Assessment Tool (CPAT, <http://lilab.research.bcm.edu/cpat/index.php>).


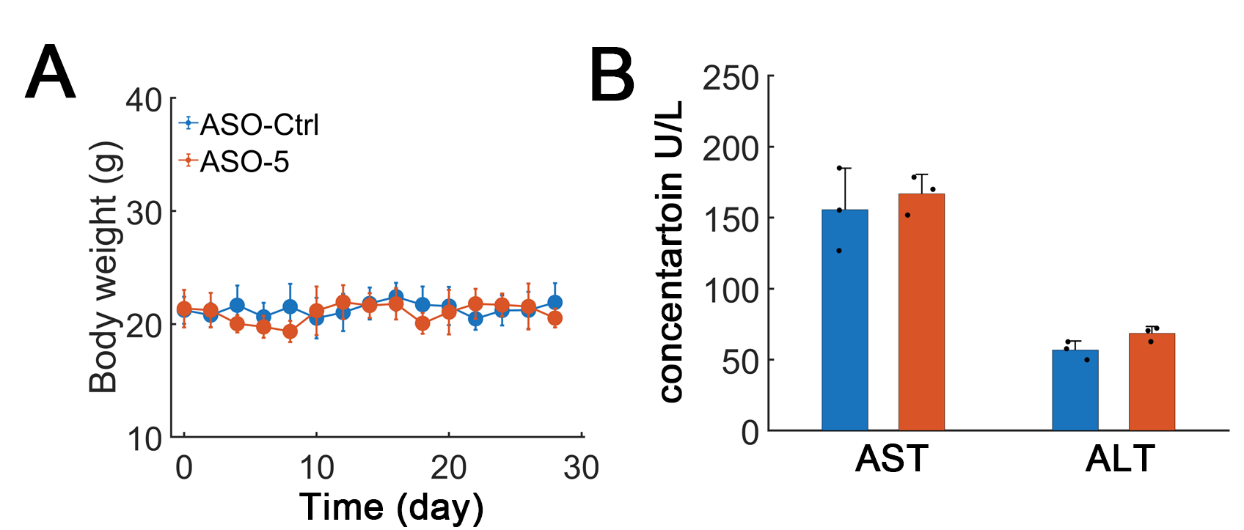


Figure S2. Effect of ASO-Ctrl or ASO-5 treatment on mice. (A) Body weights after treatment with ASO-Ctrl or ASO-5. (B) Seral AST and ALT levels in ASO-Ctrl- or ASO-treated groups (ALT: alanine aminotransferase; AST: aspartate aminotransferase). Blue: ASO-Ctrl; Red: ASO-5.

**Reference**

[1] W.J. Kent, A.S. Zweig, G. Barber, A.S. Hinrichs, D. Karolchik, BigWig and BigBed: enabling browsing of large distributed datasets, Bioinformatics 26 (2010) 2204-2207.

[2] A. Raj, P. van den Bogaard, S.A. Rifkin, A. van Oudenaarden, S. Tyagi, Imaging individual mRNA molecules using multiple singly labeled probes, Nat Methods 5 (2008) 877-879.
